# Knowledge on clinical assessment methods for using phonetics in removable prostheses among dentists in Riyadh, Saudi Arabia

**DOI:** 10.6026/9732063002001353

**Published:** 2024-10-31

**Authors:** Sara Tarek Ahmed, Jomana Abdulrahman, Arwa Mansour, Sara Abdulrazek, Alanoud Fahad, Khalid Abdulaziz Albesher

**Affiliations:** 1 Department of Prosthodontics, College of Medicine and Dentistry, Riyadh Elm University, Riyadh, Kingdom of Saudi Arabia; 2 Intern, College of Medicine and Dentistry, Riyadh Elm University, Riyadh, Kingdom of Saudi Arabia; 3 Consultant Prosthodontist, Dental Department, King Salman bin Abdulaziz Hospital, Riyadh, Kingdom of Saudi Arabia

**Keywords:** Phonetics, speech, rehabilitation, edentulous patients, removable prosthesis, prosthodontics, knowledge

## Abstract

Phonetics is a crucial part of prostheses fabrication and it plays an important role in personal and social development. This underscores the importance of understanding how various factors, including dental interventions, can influence speech quality and intelligibility. Therefore, it is of interest to assess the knowledge and clinical assessment techniques of dental students, interns and dentists in Riyadh, Saudi Arabia, to ensure their ability to deliver dental prostheses that allow clear speech. A survey questionnaire was sent to 1300 dental students and clinicians, with a response rate of 93.3% from 1213 responses collected. The questionnaire included twenty questions to assess general knowledge about phonetics in the construction of removable dentures and clinical techniques used in evaluating speech with trial dentures. The majority of participants were 5th-year dental students, followed by dental interns, general dentists, postgraduate residents, and specialists. Half of the participants, 51% of the participants, had less than one year of experience. The study found that dentists with more than five years of clinical experience have better knowledge of clinical assessment methods used to evaluate phonetics. The lowest scores were submitted by 5th-year dental students. Dentists who had the most clinical experience of 5 years or more had better knowledge and clinical assessment skills, while other dentists had the knowledge but lacked the experience. Despite phonetics being taught in dental curricula, students are unable to connect this knowledge to clinical settings. For this reason, a checklist will be made available and shared among various institutions to facilitate clinical application, improve the standard of dental prostheses provided to patients and raise patient satisfaction and quality of life.

## Background:

Speech, the fundamental means of human communication, plays a crucial role in personal and social development. It involves the use of words to convey thoughts, emotions, and perceptions articulately [1]. A person's ability to interact socially can be significantly impacted by deviations or deficits in speech features [2]. This underscores the importance of understanding how various factors, including dental interventions, can influence speech quality and intelligibility. According to previous studies, alterations in the oral cavity resulting from tooth loss can adversely affect speech quality [2]. Prosthetic restorative techniques, such as well-designed removable prostheses, can have a positive impact on speech intelligibility and articulation [3-4]. Consequently, dental students and practitioners have a moral obligation not only to restore missing teeth for improved speech but also to ensure the overall comfort and quality of life for patients receiving prosthetic restorations [5].

Despite the significance of speech in dental practice, the existing literature reveals a gap in research studying dentists' knowledge, comprehension skills and clinical methods related to speech evaluation. This study aims to address this gap by examining the practices of dental students, postgraduate residents, and dentists in Riyadh, Saudi Arabia. By evaluating their knowledge and clinical techniques, the study seeks to provide guidance for enhancing speech education in dental training programs. This may involve curriculum updates, continuing education courses, or the implementation of checklists in dental clinics to support practitioners. Ultimately, such improvements are expected to enhance dental practices and contribute to an improved quality of life for patients receiving removable prostheses. Therefore, it is of interest to assess the knowledge of clinical assessment techniques of dental students, interns and dentists in clinical practice, to guarantee their ability in delivering prostheses to patients that permit clear speech.

## Materials and Methods:

## Study design and Participants:

This cross-sectional survey study was conducted among dental students, interns and dentists in Riyadh, Saudi Arabia. The survey questionnaire was distributed electronically using Google Forms. A total of 1300 dental students and clinicians were invited to participate and 1213 responses were collected.

## Ethical approval:

The institutional review board approved this study at Riyadh Elm University with the IRB approval number "FUGRP/2023/336/1056/956".

## Inclusion and exclusion criteria:

The study included senior dental students, dental interns, senior prosthodontics postgraduate residents, general dentists and prosthodontists. Non-practicing dentists, first-year dental students, and first-year prosthodontics postgraduate residents were excluded from the study.

## Instrument validity and reliability:

The questionnaire consisted of twenty closed-ended questions divided into two domains. The first domain contained five questions aimed at assessing the general knowledge of participants regarding the role of phonetics in the construction of removable dentures. The second domain included fifteen questions related to the clinical techniques employed in evaluating speech with trial dentures. Participants were provided with three response options for each question: "I don't know", "incorrect" and "correct".

To ensure the validity of the questionnaire, it was reviewed by three experts with extensive experience in removable prosthodontics. Their feedback on the questions and the relevance of each part in measuring the intended outcomes were considered. The reliability and clarity of the questionnaire were evaluated through a pilot study involving 100 individuals from the target population.

## Patient confidentiality:

Strict measures were taken to maintain the confidentiality of participant data. The collected information was treated as confidential and was not disclosed or used without the explicit consent of the participants.

## Statistical analysis:

Statistical analysis was performed using IBM SPSS Statistics software (Version 22). Descriptive analysis was used to summarize the data, with frequency and proportions for categorical variables and mean ± SD for continuous variables. Response scores were assigned for analysis, with "1" representing correct responses and "0" for incorrect or "I don't know" responses. Sum scores for domains and total scores were calculated to facilitate comparison based on socio-demographic characteristics. As the data did not follow a normal distribution, non-parametric tests were employed. The Kruskal-Wallis test was used to compare mean response scores based on socio-demographic characteristics, with a significance level set at P < 0.05.

## Results:

The distribution of research participants according to their socio-demographic attributes is shown in Table 1. The age group of 21-30 years old accounted for 78.0% of the participants, with 31-40 years old coming in second with 19.6%, and those over 41-50 years old with 2.4% of the total. The proportion of genders was fairly equal, with 52.8% of the population being female and 47.2% being male. In terms of education, dental interns (26.5%), general practitioners (23.5%), postgraduate residents (10.8%) and specialists (6.4%) made up the largest percentage of participants (32.8%). The majority (51.0%) of those with clinical experience had less than a year's worth, followed by 1-3 years (27.3%), 3-5 years (13.9%), 5-10 years (6.4%), and more than 10 years (1.3%). The majority of participants were enrolled in or working for private dental offices (23.7%), academic institutions (3.3%), government hospitals (11.3%), private universities (30.1%), and government universities (31.7%).

The distribution of participant answers evaluating broad knowledge about the function of phonetics (Domain 1) is shown in Table 2. Ninety-five percent of participants correctly recognised phonetics as the study of human voice acoustics. In a similar vein, a sizable portion (89.2%) accurately identified how appropriate tooth alignment and denture base thickness influence sufficient speech. 85.7% of participants correctly identified the try-in visit as the preferred period for phonetic assessment. But only 48.3% of respondents correctly said that the speech adaption phase following full denture placement lasts between two and four weeks. 87.8% of subjects correctly identified the effect of unsupported lips, decreased face height, or incorrect tooth alignment on pronunciation.

The distribution of participant replies about the clinical assessment of phonetics during dental prosthesis construction (Domain 2) is shown in Table 3. The replies of the participants to particular phonetic evaluation questions are shown in the table. The proportion of accurate answers differed depending on the question. For instance, only 35.5% of participants correctly identified that the thickness of the labial flange and the anteroposterior position of anterior teeth can alter the labiodental "b," "p," and "m" sounds. Other questions had varying accurate answers, such as Class III patients having trouble pronouncing specific sounds (23.4% correct), Class I patients pronouncing the "s" sound correctly (72.0%), and Class I patients detecting an incorrect vertical dimension of occlusion (67.1% correct).

## Discussion:

This study was aimed to evaluate and compare the level of knowledge and clinical evaluation methods of speech in removable prosthesis used by undergraduate and postgraduate dental students, dental interns, and practitioners in Riyadh, Saudi Arabia. Students with different academic levels from various universities and dental specialists from different practices were included, to obtain an overall view of the level of understanding and clinical assessment methods applied by students from their first years of clinical practice and how it progresses and builds up depending on their job environment and years of clinical experience. Our study hypothesis was supported by the survey results, which revealed differences in the students' knowledge when compared to dental specialists, with multiple values suggesting inadequate knowledge of several topics. This suggests the need to update the curricula and place greater emphasis on clinical training.

In the general knowledge domain, most of the questions were answered correctly with the highest scores obtained by the dentists who worked in academics. This domain mainly discussed the general cause of improper pronunciation, how adequate speech in general is achieved by proper positioning of teeth and thickness of denture flanges and the speech adaptation period of the patient after insertion of the removable prosthesis which is 2-4 weeks. In the clinical evaluation domain, the highest scores were for question 9 in which 1028 participants (85.2%) has answered correctly, these findings agreed with the previous study by Abualsaud *et al.* [6] where the participants had the highest scores of correct answers on a different statement about the F and V sounds, which are labiodental sounds, created by upper incisors in contact with the labiolingual center of the posterior 3rd of the lower lip, also called wet-dry line [7].

On the other hand, the lowest score was obtained from question 7 were answered correctly by 152 participants (12.6%) of the statement that includes lingual positioning of the upper anterior teeth while pronouncing the s sound, whereas the rest either answered incorrectly (65.5%), or as they don't know (22.0%). One of the lower scoring questions was question 6 where 413 participants (34.2%) answered correctly, the statement suggests that whistling when pronouncing "s" sound is caused by the upper premolars obstructing the tongue, whereas (15.9%) answered incorrectly, or as they don't know (50.0%). Similarly, question 18 in a previous study [6] suggests that "whistling" is a result of posterior teeth set too far lingually, making the question one of the lower scoring questions as well with only 102 (29.7%) participants answering correctly. (21.5%) answered incorrectly, (48.8%) answered I do not know. The results of the present study showed there was a significant correlation between years of clinical experience and the level of general knowledge and clinical skills used to assess speech. Dentists with more than 5 years of clinical experience had significantly higher levels of knowledge of the clinical assessment methods used to evaluate phonetics compared to dentists with lesser years of experience. The lowest scores were submitted by 5th year dental students. This is in agreement with a previous study [6] where they found that the 5th year dental students of Imam Abdulrahman Bin Faisal University, in Dammam had less amount of knowledge compared to dental interns. Different disadvantages have been reported with cross-sectional surveys, with weakest point being that they're based on questionnaires [6, 8]. The likelihood of respondents to submit favorable or careless replies are also major drawbacks, which could lead to an exaggerated or an inaccurate/false assessment of the dental students' and dentists' knowledge [6, 9].

This study also showed that the majority of questions which were answered correctly were for the "F", "V", "S", "T", "D", "N" and "TH" sounds which their phonation was mainly affected by the anterior region of the removable prosthesis. The correct positioning of anterior teeth and thickness of the flanges/anterior palatal region of the prosthesis greatly affect the phonation of these sounds and also has a great impact on the esthetic appearance of the patient [10-12]. Nowadays, the esthetic value of the prosthesis is of utmost importance, when delivering a removable prosthesis [13-14]. Thus, greater attention and evaluation methods would be applied to achieve satisfactory results. Hence, the clinicians would be more familiar with the phonetic assessment of sounds affected by the anterior region.

## Conclusion:

Data shows that dentists who had the clinical experience of 5 years or more had better knowledge with clinical assessment skills, while other dentists had the knowledge but lacked the experience. One of the helpful techniques which could help dentists apply the knowledge they have could be by providing a checklist with simple statements to evaluate speech in removable prosthesis. It is noted that students are not able to correlate this information with the clinical practice even though the study of phonetics is included in dental curriculum. Therefore, a checklist could be provided and distributed among different institutes in order to simplify the clinical application and enhance the quality of dental prosthesis delivered to the patients in order to achieve higher levels of patients' satisfaction and quality of life.

## Figures and Tables

**Table 1:** Distribution of study participants based on socio-demographic characteristics

**Variable**	**Category**	**n (%)**
Q1. What is your age group?	21-30 years	946 (78.0%)
	31-40 years	238 (19.6%)
	> 41-50 years	29 (2.4%)
Q2. What is your gender?	Male	573 (47.2%)
	Female	640 (52.8%)
Q3. What is your educational level?	5th year dental student	398 (32.8%)
	Dental Intern	321 (26.5%)
	General practitioner	285 (23.5%)
	Postgraduate resident	131 (10.8%)
	Specialist	78 (6.4%)
Q4. How many years of clinical experience have you had?	< 1 year	619 (51.0%)
	1-3 years	331 (27.3%)
	3-5 years	169 (13.9%)
	5-10 years	78 (6.4%)
	> 10 years	16 (1.3%)
Q5. Where are you currently studying or employed?	Private dental practice	287 (23.7%)
	Government hospital	137 (11.3%)
	Academics	40 (3.3%)
	Private University	365 (30.1%)
	Government University	384 (31.7%)

**Table 2:** Distribution of participants' responses assessing the general knowledge regarding the role of phonetics (Domain 1)

**Questions**	**Responses**	**n (%)**
Q1. The study of the acoustics of the human	Correct	1154 (95.1%)
voice is known as phonetics.	Incorrect	25 (2.1%)
	I don't know	34 (2.8%)
Q2. Adequate speech is affected by proper teeth	Correct	1082 (89.2%)
positioning and denture base thickness.	Incorrect	113 (9.3%)
	I don't know	18 (1.5%)
Q3. The preferred time to assess phonetics is at the try-in visit.	Correct	1039 (85.7%)
	Incorrect	125 (10.3%)
	I don't know	49 (4.0%)
Q4. The speech adaptation period after complete	Correct	586 (48.3%)
denture insertion is 2-4 weeks.	Incorrect	267 (22.0%)
	I don't know	360 (29.7%)
Q5. Unsupported lips, altered facial height, or wrong teeth	Correct	1065 (87.8%)
positioning can all result in improper pronunciation.	Incorrect	112 (9.2%)
	I don't know	36 (3.0%)

**Table 3:** Distribution of participants' responses regarding the clinical evaluation of phonetics during the construction of dental prostheses (Domain 2)

**Questions**	**Responses**	**n (%)**
Q1. The "b", "p", and "m" sounds are labiodental. Therefore, they can be affected by the anteroposterior position of anterior teeth and the thickness of the labial flange.	Correct	431 (35.5%)
	Incorrect	234 (19.3%)
	I don't know	548 (45.2%)
Q2. Class III patients have difficulties with "p"," b", "m" and "s" sounds.	Correct	283 (23.4%)
	Incorrect	373 (30.8%)
	I don't know	555 (45.8%)
Q3. During pronunciation of the "s" sound, the incisal edges of the upper and lower teeth should come very close to each other but without contact in class I patients.	Correct	871 (72.0%)
	Incorrect	98 (8.1%)
	I don't know	241 (19.9%)
Q4. Upon pronunciation of the "s" sound, contact of the upper and anterior teeth causes "clicking", which indicates excessive vertical dimension of occlusion (VDO)?	Correct	810 (67.1%)
	Incorrect	115 (9.5%)
	I don't know	282 (23.4%)
Q5. Improper VDO can be detected by evaluating the pronunciation of the word "emma".	Correct	455 (37.6%)
	Incorrect	328 (27.1%)
	I don't know	427 (35.3%)
Q6. "Whistling" when pronouncing the "s" sound indicates obstruction of the tongue by upper premolars.	Correct	413 (34.2%)
	Incorrect	192 (15.9%)
	I don't know	604 (50.0%)
Q7. When the "s" sounds more like "sh," this is due to the lingual position of the upper anterior teeth.	Correct	792 (65.5%)
	Incorrect	152 (12.6%)
	I don't know	266 (22.0%)
Q8. Class II patients have difficulties with "s", "z", f " and "v" sounds.	Correct	343 (28.4%)
	Incorrect	339 (28.0%)
	I don't know	527 (43.6%
Q9. The upper central incisors edges of the patient must contact the vermillion border of his/her lower lip at the junction of the wet-dry mucosa to pronounce the "f" and "v" sounds.	Correct	1028 (85.2%)
	Incorrect	86 (7.1%)
	I don't know	93 (7.7%)
Q10. When the "v" sounds like an "f", it indicates that the teeth are set too high above the occlusal plane or the teeth are too short.	Correct	658 (54.5%)
	Incorrect	140 -11.6%
	I don't know	410 (33.9%)
Q11. "t", "d" and "n" are palato-lingual sounds that appear when the tongue is pressed firmly against the anterior hard palate (tongue and hard palate).	Correct	883 (73.2%)
	Incorrect	112 (9.3%)
	I don't know	211 (17.5%)
Q12. Excessive thickness of the anterior portion of the denture base of the maxillary denture would result in patients pronouncing the "m", as a "b".	Correct	418 (34.7%)
	Incorrect	156 (12.9%)
	I don't know	632 (52.4%)
Q13. Difficulty differentiating the "th" from the "t" sounds indicates inadequate interocclusal distance.	Correct	648 (53.6%)
	Incorrect	129 (10.7%)
	I don't know	432 (35.7%)
Q14. Difficulty pronouncing the "g" sound indicates excessive thickness of the denture base in the post-dam region.	Correct	476 (39.4%)
	Incorrect	148 (12.3%)
	I don't know	584 (48.3%)
Q15. Over-extension of the maxillary denture posteriorly negatively affects the "k" sound to be more like "ch".	Correct	389 (32.5%)
	Incorrect	167 (14.0%)
	I don't know	641 (53.6%)

## References

[1] Lee SY (2023). J Prosthodont Res..

[2] Bhat JT (2021). Int J Appl Dent Sci..

[3] Godbole S (2016). IOSR Journal of Dental and Medical Sciences..

[4] Kheraif AA (2012). Middle East J Sci Res..

[5] Budala DG (2023). Medicina (Kaunas)..

[6] Abualsaud R (2022). Dent J (Basel)..

[7] Mahoorkar S (2016). J Indian Prosthodont Soc..

[8] Lu CY (2009). Int J Clin Pract..

[9] Boroumand S (2008). J Cancer Educ..

[10] Hussein M, Hristov I (2020). Folia Med (Plovdiv)..

[11] Oweis Y (2022). Int J Dent..

[12] Roumanas ED (2009). J Prosthodont..

[13] Niyogi S (2021). Int J Otorhinolaryngol Head Neck Surg..

[14] Sampaio-Fernandes M (2020). Eur J Dent Educ..

